# A transcriptional-switch model for Slr1738-controlled gene expression in the cyanobacterium *Synechocystis*

**DOI:** 10.1186/1472-6807-12-1

**Published:** 2012-01-30

**Authors:** Paul Garcin, Olivier Delalande, Ju-Yuan Zhang, Corinne Cassier-Chauvat, Franck Chauvat, Yves Boulard

**Affiliations:** 1 CEA, Institut de Biologie et de Technologies de Saclay, Service de Biologie Intégrative et Génétique Moléculaire, LBI, CEA-Saclay, F-91191 Gif sur Yvette CEDEX, France; 2 CNRS, URA 2096, F-91191 Gif sur Yvette CEDEX, France

## Abstract

**Background:**

Protein-DNA interactions play a crucial role in the life of biological organisms in controlling transcription, regulation, as well as DNA recombination and repair. The deep understanding of these processes, which requires the atomic description of the interactions occurring between the proteins and their DNA partners is often limited by the absence of a 3D structure of such complexes.

**Results:**

In this study, using a method combining sequence homology, structural analogy modeling and biochemical data, we first build the 3D structure of the complex between the poorly-characterized PerR-like regulator Slr1738 and its target DNA, which controls the defences against metal and oxidative stresses in *Synechocystis*. In a second step, we propose an expanded version of the Slr1738-DNA structure, which accommodates the DNA binding of Slr1738 multimers, a feature likely operating in the complex Slr1738-mediated regulation of stress responses. Finally, in agreement with experimental data we present a 3D-structure of the Slr1738-DNA complex resulting from the binding of multimers of the FUR-like regulator onto its target DNA that possesses internal repeats.

**Conclusion:**

Using a combination of different types of data, we build and validate a relevant model of the tridimensional structure of a biologically important protein-DNA complex. Then, based on published observations, we propose more elaborated multimeric models that may be biologically important to understand molecular mechanisms.

## Background

DNA-binding proteins play a crucial role in many fundamental biological processes including transcription, regulation, as well as DNA replication and repair. Thus, a better understanding of DNA-protein interactions has both a fundamental research interest and an applied importance in medicine (development of drugs interfering with oncogene expression) and biotechnology (genetic engineering of microbial organisms).

In the past, a lot of effort has been made to understand the basic principles that govern the specificity of protein-DNA interactions. It appeared that there is no simple recognition code linking the DNA interacting amino acids of a protein with their target DNA nucleotides [1]. Furthermore, there are currently no standard methods to build a 3D-structure model for the representation of a DNA-protein complex, unlike what occurs for protein-protein interactions [2]. All current methods for predicting the structures of protein-DNA complexes use the features of the unbound protein and DNA partners and various algorithms (shape complementarity, surfaces properties, experimental contacts...) to drive the docking, and propose a model for the studied protein-DNA complexes. By contrast, in this study, we used the experimentally-determined structures of protein-DNA complexes that are presumably similar to the one we study, to build a model representation of its possible structure. For this purpose, we selected among the DNA-protein complexes available at the PDB database, those sharing secondary structure motif analogy with our protein of interest, irrespective of the sequence homology between these reference proteins and our studied protein. This strategy, aims to preserve the structural conformations required to establish the interactions between amino acids and nucleotides in the model complexes. In order to test the feasibility of this strategy, we applied it to manually build a reliable model of the complex occurring between Slr1738, an important but structurally uncharacterized member of the family of PerR transcription regulators, and its target DNA. The PerR family of regulators belongs to the larger family of bacterial FUR regulators (ferric uptake regulator), which control the responses to iron or zinc availabilities [3], for a review see [4]. PerR was initially characterized as the master regulator of the *Bacillus subtilis *responses to hydrogen peroxide [5], which regulates the anti-oxidant genes encoding the DNA binding protein MgrA, the catalase KatA, the alkyl hydroperoxide reductase AhpCF, PerR itself, and FUR, in accordance with the interplay between iron homeostasis and protection against oxidative stress. PerR is a small dimeric protein that contains two metal ions per monomer and binds to AT-rich DNA motifs of the promoter region of its target genes. One metal-binding site coordinates a zinc ion that plays a structural role, while the second site binds the regulatory metal, Fe^2+ ^(PerR-Zn-Fe) or Mn^2+ ^(PerR-Zn-Mn). PerR senses H_2_O_2 _through the Fe-catalyzed oxidation of its H37 or H91 amino acid residues, leading to dissociation of the PerR-DNA complex [6]. The recent crystallographic structures of the PerR protein, though not complexed to its target DNA, suggested possible mechanisms by which PerR undergoes similar conformational changes upon binding either Fe or Mn. The structure of the PerR-Zn protein lacking Fe [7] reveals the two CXXC motifs involved in the tetrathiolate coordination of Zn^2+ ^that stabilizes the PerR dimer in a flat conformation poorly suited to bind DNA. The structures of the fully metalated proteins PerR-Zn-Mn and PerR-Zn-Fe indicate that the binding of either Mn^2+^or Fe^2+ ^ions, likely to the same pair of regulatory sites on the dimer, lead to a caliper-like close conformation better suited to bind DNA [5,8].

PerR-like regulators occur in a wide variety of prokaryotic organisms, including cyanobacteria, which are important for the Biosphere in producing a large part of the atmospheric oxygen and the biomass for the food chain [9], and have promising biotechnological potentials [10-12]. By their nature, cyanobacteria are frequently challenged by the intrinsically related oxidative and iron stresses, as they perform the two main iron-requiring oxidant-generating processes respiration and photosynthesis [13]. In this study, we pursued the analysis of the PerR-like regulator Slr1738, which controls the responses to oxidative and metal stresses in the widely-used model cyanobacterium *Synechocystis PCC6803 *[14-16]. We identified the transcription start site and the crucial -10 promoter element for the two oppositely oriented genes *slr1738 *and *sll1621 *(*sll1621 *encodes the antioxidant enzyme AhpC (alkylhydroperoxidase [13])). We also characterized the long (33 bp) AT rich motif involved in the Slr1738-mediated repression of *sll1621*. Also interestingly, we built a 3D structural model of Slr1738 complexed with its AT-rich target DNA. This model will be of great help to decipher the molecular mechanisms operating in the tight interplay between iron homeostasis and tolerance to metal and oxidative stresses. Furthermore, starting from our protein dimer model, we also propose more speculative complex, *i.e*. oligomeric, structures (tetramer, hexamer, etc...) as possible molecular effectors of the numerous regulations controlled by Slr1738 [17].

## Methods

### Experimental work

#### Determination of the transcription start sites of the divergently transcribed slr1738 and sll1621 genes

Total RNAs were isolated from *Synechocystis *and treated with shrimp alkaline phosphatase (SAP) that does not affect full-length mRNA, which have 5'-triphosphate ends, but dephosphorylates degraded RNA, which have a 5'-monophosphate extremity. Then RNAs were treated with tobacco acid pyrophosphatase (TAP), which converts the 5'-triphosphate of full length mRNA into 5'-monophosphate, but does not modify the 5'-OH of the degraded RNA. Then the 5'-monophosphate extremity of the full length mRNA was ligated to an RNA anchor with the T4 RNA ligase, and the resulting chimeric RNA was reversed transcribed with a gene-specific primer, thus creating the first strand of cDNA. This cDNA strand was amplified by PCR using both the DNA version of the RNA anchor at the 5' extremity, and the gene specific primer at the 3' side. Finally, the PCR-amplified DNA was sequenced to determine the nature of the nucleotide immediately downstream of the DNA anchor oligonucleotide that corresponds to the transcription start site (TSS).

#### Construction of transcriptional fusions to the cat reporter gene and CAT assay

The *slr1738 *promoter region and segments thereof were amplified by PCR, using site-specific oligonucleotides that flanked the PCR DNA product with *Sna*BI blunt-ended restriction sites in such a way that all nucleotide substitutions were eliminated upon cleavage with *Sna*BI. The resulting blunt-ended promoter fragments were cloned in the unique *Sna*BI site of the pSB2A promoter probe vector [18], *i.e*. in front of its promoter-less *cat *reporter gene. The sequence of every promoter insert was verified (Big Dye kit; ABI Perkin-Elmer) before and after replication in *Synechocystis*. Then, 1-2 × 10^9 ^reporter cells grown on standard plates up to mid-log phase culture were rapidly harvested and disrupted with an Eaton press, prior to CAT assay [19]. CAT activities are the mean value of three measurements performed on two independent cellular extracts; 1 CAT unit = 1 nmol of chloramphenicol acetylated. min^-1^. mg^-1 ^of protein.

### Modeling work

#### Slr1738 monomer construction

**Figure 1:**
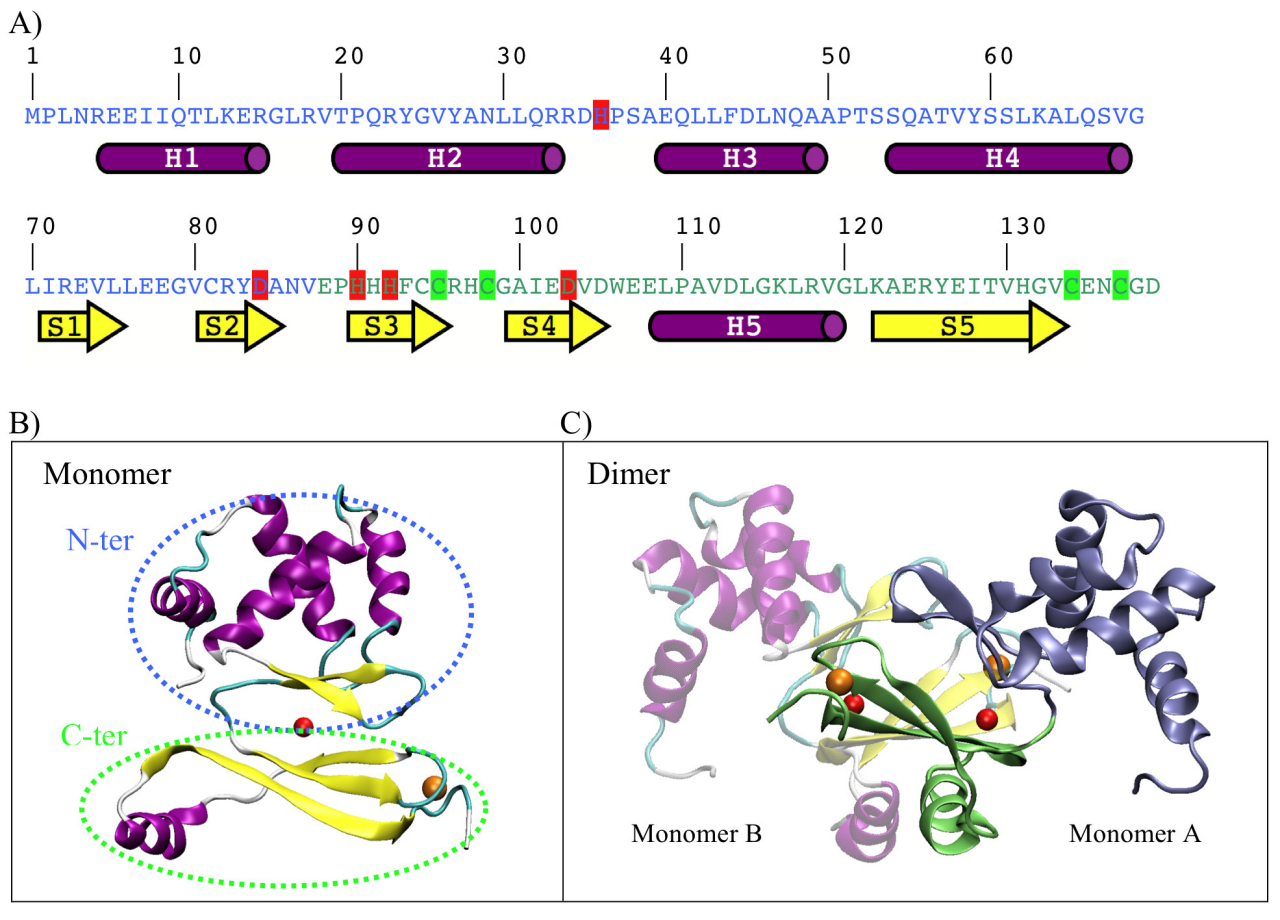
**Protein Slr1738 features**. (A) Slr1738 primary sequence (N-terminal domain in blue and C-terminal domain in green) and predicted secondary structure elements (α-helix in purple and β-strands in yellow). Metallic ligands are highlighted in red for iron and in green for zinc site. B) 3D monomeric and C) dimeric model of Slr1738 in ribbon representation (iron and zinc ions are respectively represented in red and orange van der Waals spheres).

The homology model for the monomer of the *Synechocystis *PerR-like regulator Slr1738 was obtained after sequence alignment of the structure of the closely related FUR protein of *Pseudomonas aeruginosa *(PDB ID 1MZB) [20] and built using the modeller program. Completion of the starting structure of the PerR-like model was achieved with the xLeap module of Amber 9 suite, to finally get the correct 139 residues protein sequence (Figure 1A). The final structural model of the PerR-like protein was obtained by short MD relaxation.

#### Metallic sites parametrization

Considering the nature of the amino acid ligands of the two potential metal-binding sites (using analogy to the family of FUR regulators and its PerR-type sub-family), the Slr1738 protein should likely be a PerR type containing one iron atom and one zinc atom per monomer. Zinc site forcefield parameters have been already proposed for Amber [21,22], so we used a combination of these data and of the tetrahedral geometry of a four-cysteine zinc site in a metallothionein (PDB ID 1JJD) [23] as a structural reference to set the zinc site parameters. The four C95, C98, C134 and C137 cysteines of Slr1738 likely involved in metal coordination (determination by CxxC pattern analogy to PerR regulator) are covalently bonded (*i.e*. explicit bonded terms in the forcefield) to the zinc atom via their sulfur atom.

The iron site is not sufficiently defined for a rigorous semi-empirical treatment at simple atomic resolution. Iron should be in its ferrous state [24] and penta- or hexa-coordinated to histidine (via deprotonated pyrrole nitrogen) and aspartic acid residues [8]. We chose to model the metal ion as a hard sphere with a +2.0 raw charge and a 1.2 Å van der Waals radius. The coordination site was set via distances harmonic constraints (100 Kcal.mol^-1^.Å^-1^) between iron and its amino acid ligands. Despite inducing potential local distortions, the overestimation of the charge should prevent dissociation of the metal ion from its binding site and preferentially stabilize the folded conformation. In our initial model, iron presents a tetrahedral geometry (H32, E80, H89 and E100 in the *Pseudomonas aeruginosa FUR *regulator are respectively H36, D84, H92 and D103 in the *Synechocystis *Slr1738 regulator) resulting from the experimental Fe/Zn-substituted structure. Bipyramidal base-square geometry is reached with histidine H90 residue coordination and the addition of a water molecule that could be crucial for the function of the protein [6].

#### Molecular mechanics and molecular dynamics (MD) simulations

All simulations have been performed using Parm99 forcefield and programs of Amber 9 suite [25]. Molecules were neutralized with Na^+ ^ions and placed in TIP3P water boxes for explicit solvation. After energy minimisations, models were used to initiate MD simulations. Short MD simulations (5ns for the production period) were principally used to allow accelerated geometry optimisation after structure modifications. This provided also qualitative information about stability of the models and the flexible regions of a structure. Final models were obtained after geometric average on the whole stabilized trajectories followed by energy minimization in a solvent box.

#### Energy association and contact surface calculations

Binding free energies between DNA and proteins molecules were estimated using the MM-PBSA method [26]. This method was used with success for several biological protein-ligand complexes [27-29] though the final values need to be interpreted with caution due to approximations in entropic contributions. To avoid such problems, we compared only the relative, not absolute, values between the different complexes. Each complex used was rigorously comparable in terms of number of atoms. Contact surface interactions were computed with the MSMS program [30]. It gives access to the solvent accessible surface area (SASA), which was used to calculate the contact surface values between a receptor and its ligand by using the formula reported bellow.

$$CS=\frac{SASA_{rec}+SASA_{lig}-SASA_{cplx}}{2}$$

#### Three dimensional construction of the [(Slr1738-Zn-Fe)_2_-DNA] complex

##### Choice of the template structure

We built a Slr1738-DNA complex by structural analogy with the 239 PDB structures of DNA-protein complexes involving transcription factors. Among those, we considered only the DNA binding proteins harbouring a presumptive helix-turn-helix (HTH) motif predicted by the Pfam database. HTH motifs are known to vary widely in sequence over the whole DNA-binding domain and their relationships can often only be based on structure similarity [31]. Finally, we retained 4 structures [32-35] because they complied with the following qualitative criteria (Additional file 1 Table S1) found in FUR proteins [20]: 1) the size of the double stranded DNA target site is ≥ 20 bp; 2) to be complexed with DNA the protein must be homodimeric; 3) the HTH motif (*ca*. 25 residues in length) must be followed by two anti-parallel β-strands. For information, the sequence alignment of the HTHw motifs is given in Additional file 2 Figure S1; 4) the resolution of the experimental X-ray structure must be good. Note that as observed for the FUR dimer of *Escherichia coli *[36], the two recognition helices H_4 _are almost perpendicular and thus should be positioned on both sides of the DNA global axis, and not on the same side as occurs with parallel helices (Additional file 3 Figure S2).

##### Fitting procedure

Using successive energy minimisation steps, the Slr1738 monomer was fitted onto each of the 4 reference complexes by positioning the H_4 _recognition helix in the major groove of the target DNA. This global protein-DNA association driven by distance restraints was performed with frozen Slr1738 secondary structure and ended after reproducing the geometry observed in the selected structural patterns. We tested four kinds of superposition motifs to select the best one enabling the largest surface of protein/DNA contacts as shown in Additional file 4 Table S2. We note that the contact surface differences between the template structures reflect differences in the DNA sequences. Then, we replaced the template nucleotides in the model by the Slr1738 DNA-binding sequence while maintaining the phosphodiester backbone of DNA. After geometry optimization, we selected only three structures on the quality of their surface contacts and association energies (Table 1). The final three models were derived from different PDB structures (1SAX, 1U8R and 1Z9C).

**Table 1:** DNA-protein complexes parameters of the structures built with the target DNA sequence.

	Superposition type			
	
	H4S1S2		H3H4S1S2	
	
PDB name	Contact surface (Å^2^)	Association energy with DNA (21 bp)	Contact surface (Å^2^)	Association energy with DNA (21 bp)
1C0W	900	35.4	888	19.03
1SAX	890	23.92	**946**	13.18
1U8R	949	25.88	885	**7.18**
1Z9C	**1006**	**-6.82**	939	11.14

Association energies and contact surfaces of 3D models with different secondary structure motifs superpositions for the construction of the DNA-(Slr1738)_2 _complex. Best values are in bold.

##### Closure of the structure

**Figure 2:**
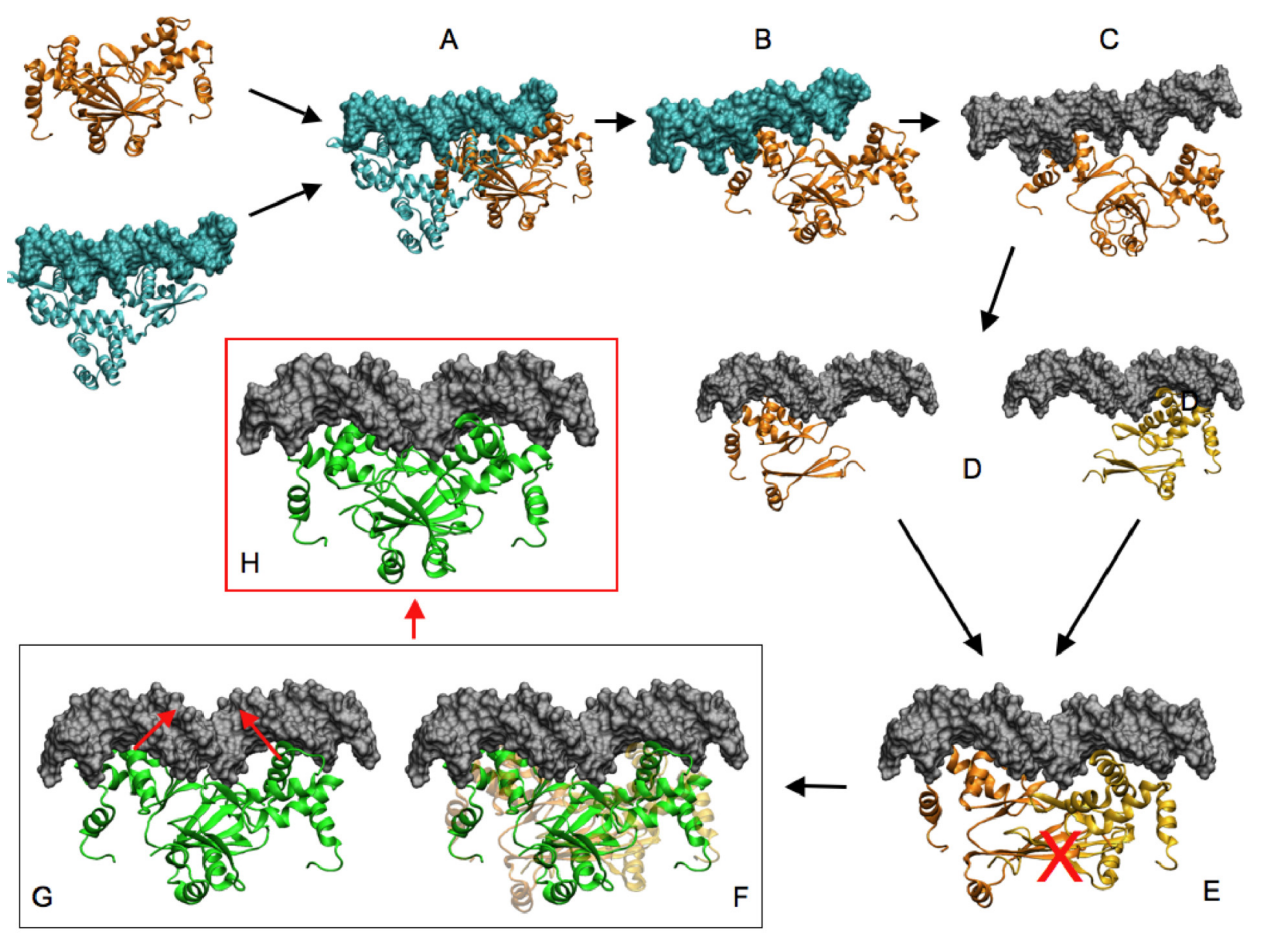
**Different steps of the 3D model construction of DNA-(Slr1738)_2 _complex**. A - Superposition of [protein-DNA] X-ray structure with (Slr1738)_2 _based on recognition helix H4 of monomer A. B - Structure of (Slr1738)_2 _docked to X-ray DNA (monomer A is well-positioned). C - DNA sequence modification. D - Structure of the Slr1738 monomer A (orange) well docked to the DNA molecule and ideal positioning of the monomer B (yellow). E - Matching on DNA sequence of both structures in D which give rise to a bad dimerization interface for Slr1738. F - (Slr1738)_2 _with a well defined dimerization interface, in green, matched on the protein structure E, in light orange. G - Minimization protocol consisting in application of harmonic distance restraints on both the DNA recognition helices. H - Final structure.

The final step of our protocol was to close the protein-DNA complex by positioning the second monomer of Slr1738 onto its DNA target. This was done using a two-step method. First, we matched the dimer Slr1738 structure to the protein/DNA complex where both Slr1738 monomers were properly positioned. In this way, we conserved the integrity of the Slr1738 protein structure and the recognition helices remained close to their optimal positions. Second, we applied distance restraints derived from structural HTHw patterns on both monomers in order to insert the Slr1738 recognition helices into the major groove of DNA. The integrity of the sugar-phosphate DNA backbone, the dimer interface and protein secondary structures was maintained under harmonic restraints throughout the procedure. The internal energies of the complexes were finally minimized with decreasingly harmonic restraints to ensure a smooth transition of the atomic system toward a relaxed configuration. The procedure was completed with short MD simulations in solvated and neutralized conditions. Contact surfaces and the association energies of the three best-predicted models are presented in Table 2. Structures at different steps of our construction strategy are presented in Figure 2.

**Table 2:** DNA-protein complexes parameters of the final structures.

Structure name	Contact surface (Å^2^)	Association energy with DNA (kcal/mol)
1SAX (H3H4S1S2)	**1852**	**36**
1U8R (H3H4S1S2)	1083	51.23
1Z9C (H4S1S2)	1230	50.45

Association energies and contact surfaces of 3D models obtained for the final structures of DNA-(Slr1738)_2 _complex. Best values are in bold.

## Results and Discussion

### *In vivo *analysis of the slr1738 and sll1621 divergent promoters: evidence for repression by Slr1738 and role of its AT-rich DNA binding motif

**Figure 3:**
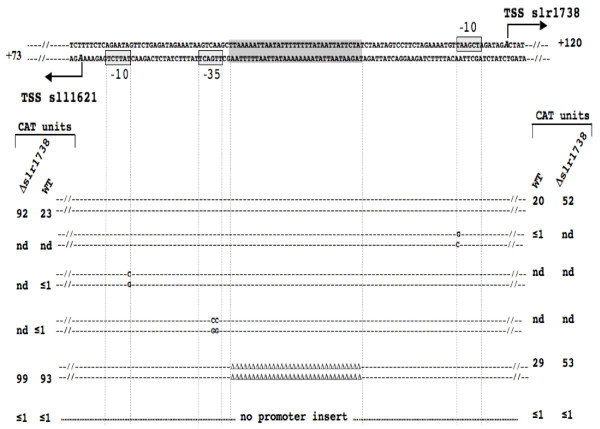
**Mutational analysis of the *slr1738 *and *sll1621 *promoter region transcriptionally fused to the *cat *reporter gene**. The transcription start-sites (TSS, + 1) are represented as bent arrows pointing into the direction of the transcription of *slr1738 *(top DNA strand) and *sll1621 *(bottom DNA strand), respectively. The length of the cyanobacterial DNA segment between the TSS and the *cat *reporter gene is indicated as +120 (slr1738-cat fusion) and +73 (Sll1621-cat fusion). Nucleotide substitutions or deletions in the promoter sequences are written in bold upper cases or represented with triangles, respectively. The -35 and -10 promoter elements are boxed and shaded in gray, like the AT-rich Slr1738-binding region (Figure 5). The CAT activities determined in wild-type or Δslr1738 mutant are the average values calculated from at least 3 independent experimental repeats (standard deviations were less than 10% of sample averages). The present data indicate that this AT-rich Slr1738-binding region operate in the Slr1738-mediated negative regulation of the sll1621 promoter activity.

We pursued the analysis of the PerR-like regulator Slr1738 that controls the responses to oxidative and metal stresses in the model cyanobacterium *Synechocystis PCC6803 *[14-16]. It was reported [37] that the deletion of *slr1738 *increases the expression of the two oppositely-oriented genes http://genome.kazusa.or.jp/cyanobase*slr1738 *itself and *sll1621*, which encodes the anti-oxidant peroxiredoxin enzyme AhpC. It has also been shown that the Slr1738 protein binds the 300 bp *sll1621-slr1738 *intergenic region [14-16], which possesses a long (30 bp) DNA motif containing only A and T nucleotides (the AT-only motif). Together, these findings suggested that Slr1738 represses both the *slr1738 *and *sll1621 *promoters. To test this interpretation, and the possible role of the long AT-only DNA motif occurring in the *sll1621-slr1738 *promoter region, we performed the following experiments. We cloned the 300 bp *sll1621-slr1738 *promoter region, in both orientations relative to the promoter-less *cat *reporter gene of our promoter-probe plasmid vector pSB2A which replicates in *Synechocystis *at about 10 copies per cell, *i.e*. at one copy per copy of the polyploïd chromosome [38]. This generated the reporter plasmids psll1621-cat and pslr1738-cat, which replicated stably in *Synechocystis*, as expected (data not shown), where they directed similar level of *cat *expression (Figure 3). As the usual control, we verified that the empty pSB2A plasmid carrying no promoter insert produced no CAT activity. Collectively, these data showed that the *sll1621 *and *slr1738 *promoters have similar strengths, which resemble those of other *Synechocystis *genes we previously studied with pSB2A [39] and references therein.

We and others [17] tried to find consensus sequences in the promoter regions of the wealth of genes presumably regulated by Slr1738. However we found none (Additional file 5 Figure S3), as occurred in the case of other PerR-like regulators [4,5,40], suggesting that particular DNA structures rather than defined nucleotide motifs in the target DNA govern PerR regulation of its target genes. This context encouraged us to perform a mutational analysis of the *slr1738 *and *sll1621 *promoters to identify their *cis*-acting promoter elements. In the *slr1738 *promoter, we studied the 5'-TAagcT-3' hexamer that matches the canonical -10 box of σ70-type *Escherichia coli *promoters [41] in both sequence (5'-TATAAT-3') and position (-12 to -7) from the transcription start site, which we presently mapped with the classical 5' RACE technique [42] that works well in *Synechocystis *[43]. It is the A nucleotide we noted as +1, which is located 131 bp upstream the *slr1838 *start codon (see Additional file 6 Figure S4). Consistent with its identification as the -10 promoter box, we found the 5'-TAagcT-3' element to be crucial to *slr1738 *transcription. Indeed, the transversion mutagenesis of its proximal T nucleotide (5'-TAagcT-3' to 5'-GAagcT-3') completely abolished *slr1738 *promoter activity (Figure 3), as occurred with the -10 promoter boxes of other *Synechocystis *genes [19,39,44-46]. By contrast, no sequence resembling a -35 promoter box (5'-TTGACA-3') was found upstream of the *slr1738 *-10 box, either at 17 bp (*i.e*. the canonical *Escherichia coli *spacing [47]) or at 30 bp (*i.e*. the distance sometimes encountered in *Synechocystis *[19,44,45]). The absence of a -35 promoter box in a *Synechocystis *promoter is not unprecedented as we previously showed that both the *lexA *and *recA *genes are well expressed from -35-less promoters [39]. Furthermore, as σ70-type promoters lacking a -35 box often possess an "extended -10 box" (5'-TGnTATAAT-3') mediating all contacts with the RNA polymerase σ70 factor [47,48], it is worth noting that the *slr1738 *promoter harbors such an "extended -10 box" (5'-TGnTAagcT-3'), like the *Synechocystis secA *promoter [19]. In contrast, the *sll1621 *promoter appeared to possess the two canonical boxes, *i.e*. a -10 element (5'-TAttcT-3'; Figure 3) located 6 nucleotides upstream of the transcription start site (the A nucleotide noted as +1, we found to be located 73 bp upstream the ATG start codon; see Additional file 6 Figure S4) and a -35 (5'-TTGACt-3') box located 17 nucleotides upstream of the -10 element.

We also introduced and tested the slr1738-cat and sll1621-cat reporter plasmids in the Δslr1738 deletion mutant we previously constructed [14]. We found the *slr1738 *promoter to be more active in the absence of the Slr1738 protein, demonstrating that Slr1738 is an autorepressor (Figure 3). Similarly, the *sll1621 *promoter appeared to be more active in the absence of the Slr1738 protein (Figure 3), demonstrating that the Slr1738-mediated downregulation of the *sll1621 *gene [14-16] is exerted at the level of its promoter activity. Furthermore, we also found that the above-mentioned long (33 bp) AT-rich motif, which we anticipated to interact with Slr1738, truly operates in the Slr1738-mediated repression of the *sll1621 *promoter (Figure 3).

### Structure modelisation of (Slr1738-Zn-Fe)_2 _protein complexed to DNA

At the beginning of our present study of the PerR-like regulator Slr1738, no structure of the metal-containing form of a PerR regulator was available in data banks. Therefore, starting from the Slr1738 primary sequence, (Figure 1A), we built its tertiary structure using homology modelling methods, energy minimization and short MD simulation. Psi-Blast analysis showed that Slr1738 exhibits 21% sequence identity (37% for homology) with the *Pseudomonas aeruginosa *FUR protein for which a crystal structure was available (PDB ID 1MZB) [20]. Based on this and other findings and on our metal titration experiments (data not shown), we completed the two Slr1738 metal binding sites with both a zinc ion (Zn^2+^) and a ferrous ion (Fe^2+^). These two metal sites were described as essential for the folding and activity of genuine PerR and FUR regulators [4,6-8,49]. In our Slr1738 model, highly conserved cysteines (C95, C98, C134 and C137) operate in the coordination of the crucial zinc atom (Additional file 7 Figure S5A). The iron-binding site displays a hexa-coordinated geometry (H36, H90, H92, and D84, D103 and a water molecule) and the anchoring of the N-terminal domain of Slr1738 on its C-terminal domain results essentially from H36-Fe coordination. However, as previously observed [15], we found experimentally that iron-less protein samples of Slr1738 were still able to bind DNA, unlike what was observed for the PerR protein where iron is crucial to DNA binding [6,8].

The resulting 3D model of Slr1738 comprises two well-defined domains (Figure 1B). The N-terminal domain from amino acids 1 to 84 contains four α-helices (H_1 _to H_4_) and a typical helix-turn-helix motif involving helices H_3 _and H_4 _that is responsible for the binding to DNA [50]. The helix H_4 _is the recognition helix that interacts specifically with the major groove of DNA. The two H_3 _and H_4 _helices are followed by two anti-parallel β-strands (S_1 _and S_2_) that formed a winged helix-turn-helix motif (wHTH) [51]. The C-terminal domain of Slr1738 from amino acid 85 to 139 comprises three β-sheets (S_3 _to S_5_) and one α-helix (H_5_) involved in the dimerization of Slr1738.

The Slr1738 dimer model we propose (Figure 1C) was first built from the *Pseudomonas aeruginosa *FUR structural information of Pohl et *al. *[20]. Our model is consistent with previous findings showing that the active form of FUR-type regulators is a dimer [52,53] and that Slr1738 too was proposed to bind to DNA as a dimer [15]. We then refined our model with the structure of the *Bacillus subtilis *PerR protein (PDB ID 2FE3 without Fe and 3F8N with Mn replacing Fe) [7,8]. The final RMSD values for the heavy atoms of the backbone were about 15 Å and 4 Å respectively for the refined structures, demonstrating the great role of iron in modifying the global folding. The zinc metal ion appears to be indispensable for structuring the C-terminal domain to enable dimerization [49]. The interactions involved at the interface of the dimer structure are depicted in Additional file 8 Figure S6.

Currently there is no 3D structure in the PDB database of a FUR/PerR-like regulator complexed with DNA. Therefore, we developed an approach, detailed in the Methods section, based on experimental 3D structures deposited in databases for predicting the structure of protein-DNA complex. Our model is consistent with the above-mentioned findings that Slr1738 binds to the 310 bp-long promoter region of the divergent genes *sll1621 *and *slr1738 *that contains a long AT-rich motif important for Slr1738-mediated regulation (Figure 3), which harbors a central symmetry for double strands, *i.e*. an ideal feature for binding a dimeric protein acting like pliers. Consequently, we chose a symmetrical 25 bp AT-rich DNA fragment for our modelling purposes, in agreement with the fact that the well-defined FUR-binding DNA sequences usually contain AT-rich palindromes [54]. We obtained the protein-DNA complex by fitting the (Slr1738)_2 _model onto well positioned monomers using a strategy based on structural analogy with experimental structures, *i.e*. the recognition helices of the HTH motif being inserted into the major groove of target DNA. The selection of the final model was based on both structural criteria and energy calculations. Our best complex model is the one based on the structural pattern derived from 1SAX.

### Considerations of DNA conformation

In our approach, we did not take into account the DNA structural specificities possibly imposed by the oligonucleotide sequence. Indeed, we kept the DNA backbone structure of the template model to maintain the structural conformations occurring at the protein-DNA interface. Hence, we selected the template structures based on the wHTH motif identification of the Slr1738 protein. This choice was dictated by our long-standing expertise concerning DNA structure [55-59]. DNA molecules are very flexible and they can adopt many local conformations [60] depending on both their nucleotide sequence and the physico-chemical environment, as discussed in a wealth of papers. As discussed above, in absence of a genuine consensus sequence for the binding of Slr1738 onto DNA, we used its AT-rich regulatory element emerging from our work (Figure 3). Such an AT-rich DNA sequence may of course adopt particular local conformations that are not easy to predict and integrate in a 3D structure. Nevertheless, we note that: 1) the final structure was minimized in allowing DNA to relax; 2) MD simulations were performed to allow the structure, and thereby the DNA molecule, to explore 3D space; 3) the four 3D structures retained for the final selection (Table 1) are very different in terms of the AT content of the target DNA sequences, it varies from 48% for 1U8R to 86% for 1Z9C. The template structure we finally retained, *i.e*. 1SAX, is 68% AT-rich. Furthermore, all these DNA structures, analyzed with the 3DNA program [61], were found to adopt a globally B-form without bending in spite of their widely different AT contents; 4) it remains difficult nowadays to predict local DNA conformations from nucleotide sequences, unlike the situation of proteins in which the secondary structure is predictable from their primary sequence. Thus, we can obviously be more confident in selecting a protein template than a DNA template.

### Structural analysis of the complex formed by the Slr1738 dimer and its DNA target

Recognition of DNA sequence is mediated by both direct interactions between amino acids and the bases in the major DNA groove (direct readout) and by contacts with DNA backbone (indirect readout). Specific interactions between Slr1738 and its target DNA concerned essentially the residues of the top of the recognition helices and *ca*. ten bases. Even though the dimeric form of Slr1738 docks an oligonucleotide of 25 bp in length, each Slr1738 monomer specifically interacts with only 5 bases in a symmetric way. The interactions are schematically represented (see Additional file 9 Figure S7) inside a simple 7-1-7 bp DNA motif, the minimal recognition motif for FUR binding [62].

**Figure 4:**
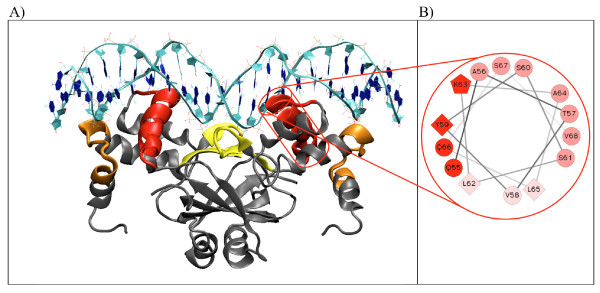
**DNA-(Slr1738)_2 _complex structure properties**. A) 3D structure of DNA-(Slr1738)_2 _complex showing six contact regions between the protein (in grey) and the DNA molecule (in cyan). For each monomer, the predicted contact regions are the loop between H_1 _and H_2 _(in orange), the helix H_4 _(in red) and the loop between S_1 _and S_2 _(in yellow). B) Helical wheel representation of the recognition helix (H_4_) of the monomer A. Three faces with different properties are presented. In soft pink are shown the hydrophobic residues turn toward the protein. In dark pink are shown the small residues facing the DNA strand 2 and in red are represented the large and charged residues in interaction with the DNA strand 1. Hydrophilic residues are present as circles, hydrophobic residues as diamonds and potentially positively charged as pentagons http://rzlab.ucr.edu/scripts/wheel/wheel.cgi.

The final model of the complex predicts that there are six contact regions between the Slr1738 dimer and its target DNA, and each monomer operates in three of them via its three sub-regions, namely: (i) the loop between H_1 _and H_2_, (ii) the α-helix H_4 _and (iii) the loop between S_1 _and S_2 _(Figure 4A), which are discussed below.

*(i) The loop between H_1 _and H_2_*. This region of Slr1738 comprises 11 amino acids including 4 positively-charged residues (^13^**K**E**R**GL**R**VTPQ**R**^23^). Two of them (R18 and R23) are presumably involved in electrostatic interactions with atoms of the negatively-charged phosphate groups of the DNA backbone in the minor groove. Sequence alignments of FUR-like proteins show that K13, R18 and R23 are highly conserved, especially among cyanobacterial Slr1738 orthologs (Additional file 7 Figure S5). This first region establishes only non-specific interactions with DNA in that there is no contact of protein side-chains with DNA bases. We propose that these long-range electrostatic interactions operate in the pre-orientation of the DNA-binding protein domain to facilitate protein/DNA interactions and in the stabilization of the resulting Slr1738/DNA complex that precedes the sequence selective interaction mediated by the H_4 _protein helix.

*(ii) The H_4 _recognition helix*. H_4A _and H_4B_, the respective recognition helices of monomers A and B are inserted into the major DNA groove where they likely act as a pair of pliers. A detailed description of the H_4 _helix is really informative in this respect. H_4 _comprises 14 amino acids (residues 55-68) organized in three different sides (Figure 4B). The first side gathers the hydrophobic residues (V58, L62 & L65) lying in direct contact with numerous hydrophobic residues of the three other helices of the N-terminal domain, thereby forming a compact hydrophobic cluster. The second side, composed of 7 small residues (A56, T57, S60, S61, A64, S67 & V68), is close to the DNA strand 2. The third side comprising bulky and charged residues (Q55, Y59, K63 & Q66) faces the DNA strand 1. The residues in close vicinity of DNA bases are mainly those of the upper part of the helix which are localized in sides two and three: Q55, A56, T57, Y59, S60 and K63. Sequence alignment of FUR proteins shows that the DNA recognition helix always contains a conserved amino acid motif (A)TVY or (A)TIY where the tyrosine is important for DNA binding [36]. For Slr1738 the H_4 _helix also has four hydroxyl-containing residues (serine and threonine residues), a feature possibly important because this DNA contact region seems to be the only one involved in sequence recognition.

*(iii) The loop between S_1 _and S_2_*. The third region of Slr1738 in contact with DNA contains three negatively-charged glutamic acids (^73^**E**VLL**EE**GVC^81^) that may interfere with the approach of the S_1 _and S_2 _anti-parallel strands to the negatively-charged DNA backbone. However, the presence of divalent cations such as Mg^2+ ^can bridge interactions between these negatively charged residues (phosphate group and glutamic amino acid) allowing the approach of the S_1 _and S_2 _anti-parallel strands. In our model, E78 is very well positioned to form such an electrostatic bridge. Furthermore, we know that the iron-binding regulatory site allows proper folding of Slr1738. In particular, its coordination by H36 induces correct contacts between the N-terminal and C-terminal domains. Thus, the modification of this site by oxidation could break the link between the N- and C-terminal domains, thereby preventing the damaged protein from binding DNA [6]. Consequently, we propose that the negatively-charged amino acids of this DNA-binding region participate via strong electrostatic repulsions with the DNA backbone to unlock Slr1738 by inducing a rotation of its N-terminal domain.

During the MD, the three contact zones described above show different behaviours. The first one, the loop between H_1 _and H_2_, remains stable thanks to the presence of the positively charged residues R18_AB_, Q22_AB _and R23_AB _which establish strong electrostatic interactions with the phosphate atoms of the target DNA that were maintained during the whole simulation. Thus, the dimeric form of Slr1738 makes six non-specific electrostatic interactions that maintain the integrity of the DNA-protein complex. The second region comprising the recognition helix H_4 _of each monomer exhibited a non-symmetrical behaviour for each monomer, in agreement with the fact that the H_4A _and H_4B _helices interact with non-similar target DNA sequences. In our model structure, only the H_4A _helix of the monomer A of Slr1738 has its tyrosine residue at a correct distance to the pair of thymines of the target DNA, which are known to interact strongly with tyrosine [33,36]. The alcohol group of the tyrosine is involved in a hydrogen bond with the phosphodiester backbone while its aromatic cycle is facing the methyl groups of the adjacent thymines. Moreover, the recognition helix and the turn preceding it contain five serines and two threonines, the repetition of which might operate in sequence specific recognition. Indeed, we noticed during MD simulations that serine or threonine could contact a thymine residue via a specific dual interaction. First, the close vicinity of the methyl group of thymine and the CH_2 _group of serine or the CH_3 _group of threonine allows the formation of a long-range hydrophobic interaction. It can also form a hydrogen bond between the alcohol group of serine or threonine and the ketone group of the thymine. Concerning the third Slr1738-DNA contact region that corresponds to the loop between S_1 _and S_2_, we observed no significant difference in the mobility of the Slr1738 protein bound or unbound to DNA. The three glutamic acids in this region induce the DNA to move slightly away from the antiparallel strands S_1 _and S_2_.

### Multimer complex hypothesis

The FUR box consensus sequence classically defined as a 19 bp inverted repeat sequence [63] binding a dimeric FUR protein is regarded by some authors as a 15 bp region with a 7-1-7 motif [62] binding a tetrameric (dimer of dimers) FUR. It was also shown that FUR does not bind to PerR boxes though they share with FUR boxes six identical nucleotides within each heptamer. A model of two *Pseudomonas aeruginosa *FUR dimers binding a canonical B-DNA was also proposed by Pohl et *al. *[20] to take into account that multiple FUR proteins protect a larger DNA region (at least 27-30 bp) than a single FUR dimer (around 20 bp). In this model, the two FUR dimers are located on opposite sides of the DNA molecule. Escolar et *al. *[64] have reinterpreted the 19 bp consensus FUR binding site as an array of three repeats of the invariable 6 bp GATAAT sequence in *Escherichia coli *while *in vitro *gel shift and DNase footprinting assays led Lavrrar *et al. *to propose that three FUR dimers (hexamer form) may bind to the 19 bp FUR box [65,66]. The possible occurrence of different forms of FUR and PerR regulators binding with different affinities onto their target genes may explain why some of them are not always co-regulated, depending on the environmental conditions.

Considering these interpretations and our 3D model, we calculated the Slr1738-buried surface of DNA with the NACCESS program [67] and found that the PerR-like regulator Slr1738 likely protects 21 bp of its target DNA, a value close to those mentioned above for FUR/PerR.

Also interestingly, while Slr1738 represses both *slr1738 *and *sll1621 *in normal conditions (see above), these two genes are not always co-regulated [68] since, for instance, *slr1738 *but not *sll1621 *is regulated positively by cadmium [14]. Consequently, by analogy with FUR and PerR regulators, we decided to explore the idea that several dimers of Slr1738 could bind to the *sll1621-slr1738 *promoter region with different patterns, thereby accounting for the similar or different regulation of the *slr1738 *and *sll1621 *genes depending on the stress. This idea was reinforced by the observation that Slr1738-like metalloregulators may occur as multimers (dimers, trimers or tetramers) under different redox conditions [69]. Therefore, we built different 3D structures of the Slr1738/target DNA complex by changing the number of and/or spacing between the Slr1738 dimers bound to the DNA. In practise, we tested all possibilities of DNA-binding of Slr1738 tetramers (dimer of dimers) by fixing the first dimer and moving the second one along the DNA with a one base pair increment so as to steadily increase the spacing between the dimers. This strategy enabled us to (i) structurally validate the different models; (ii) characterize the contact surfaces between each dimer; and (iii) examine more complex multimeric models. As expected, the interaction surface between the DNA and the proteins calculated for our models varies significantly when changing the spacing length between dimers (Additional file 10 Table S3). The DNA overlapped surface globally decreases when the spacing between dimers increases, until it reaches a stabilized value around 3300 Å^2^. Obviously, the hypothetical tetrameric models corresponding to a dimer spacing of 1, 2, 8, 9, 10 or 11 bp are unrealistic because the resulting large surface overlapping between Slr1738 dimers would generate severe steric clashes. Others model combinations without steric clashes can be divided in two groups. The first group comprises the tetramers with no contact surface between its dimers, as occurs in the 5 bp spacer model for the FUR regulator [20], and in models with spacing of 4, 14, 15, 16, 22 bp or more. The second group includes models with a spacing of 3, 6, 7, 12, 13, 18, 19, 20 or 21 bp for which we observe a contact surface between the dimers. With our model of (Slr1738)_2_, 21 bp is the maximum offset allowing inter-dimer contacts. Among all these favourable combinations, offsets of 3 and 12 bp show the highest contact surface between dimers, possibly involving a more stable tetrameric complex. However, the 6 bp-spacing model may have biological relevance, though the contact surface between dimers is smaller than in other constructions. This particular model, where the protein tetramer contacts both faces of the DNA helix, is fully compatible with the 7-1-7 inverted repeats of the *Escherichia coli *FUR target DNA, as well as the DNase I footprinting and gel shift evidence of the existence of an overlapping between *Escherichia coli *FUR dimers bound to DNA [65,66].

**Figure 5:**
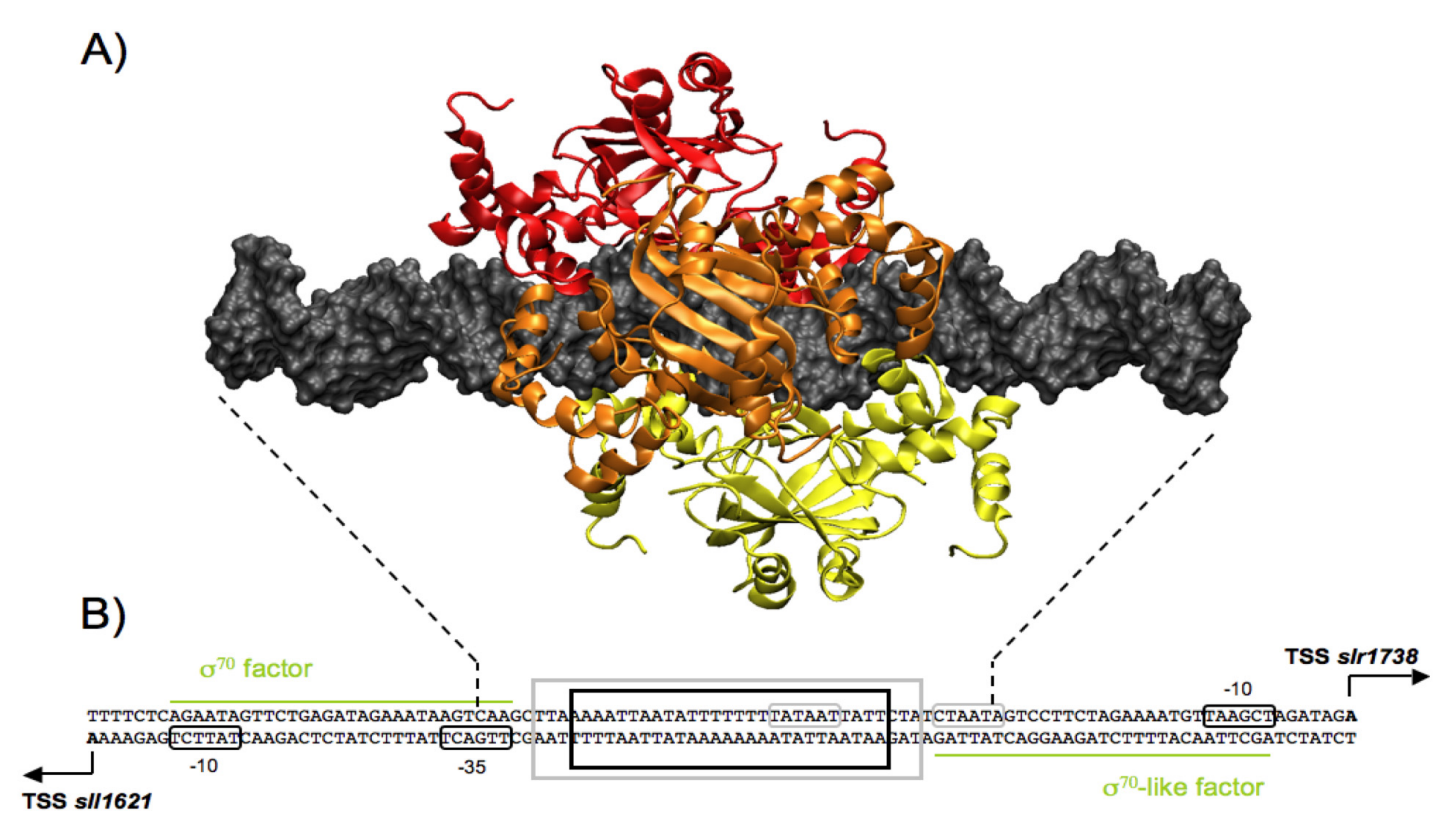
**3D structure of the hexameric model**. A) 3D hexamer structure binds to a 44 bp DNA and builds with a spacing of 3 bp between each dimers. B) Schematic representation of the DNA intergenic region between *sll1621 *and *slr1738 *genes protected by Slr1738 hexamer binding. The transcription start site, containing the promoter sequence and both -10 and -35 boxes, are reported for each gene. Dotted lines mark the boundaries of DNA fragment in the model. Black bold frame indicates the 27 bp region protected by the Slr1738 hexamer in the case of a 0-3-6 model, grey bold frame indicates the 33 bp region protected in the case 0-6-12. Green lines represent the approximate DNA recovering region by sigma factor protein that is necessary for RNA polymerase recruitment.

**Figure 6:**
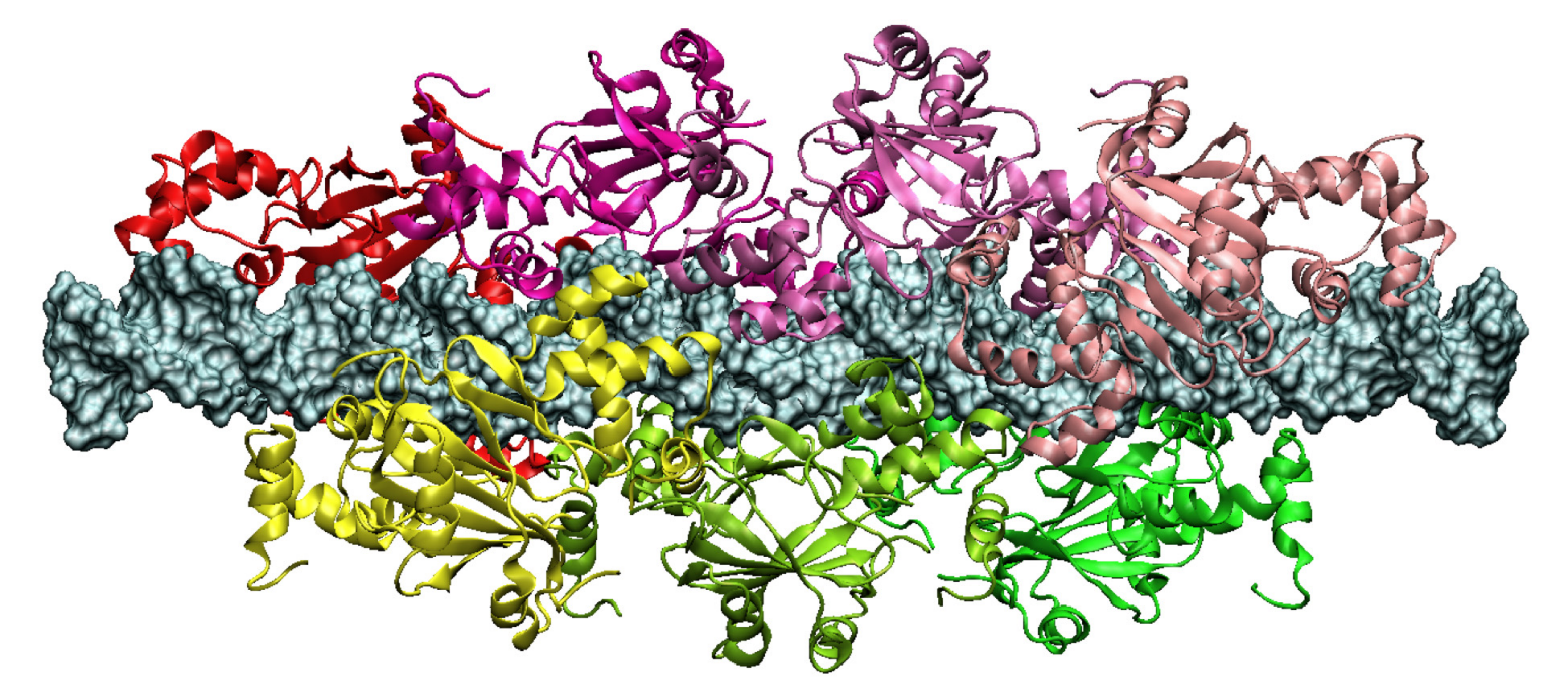
**Polymerization of (Slr1738)_2 _along DNA molecule**. Multimer 3D structure showing the polymerization of seven dimers of Slr1738 along DNA. In this figure, each dimer is separated by 6 bp from the previous one (0-6-12 model).

We also constructed more complex structural systems describing higher order multimeric regulators *i.e*. hexamers and more, and found two structures that may account for some biological observations. The first multimer structure is a hexamer composed of three dimers with a spacing of either 3 bp (0-3-6 model) or 6 bp (0-6-12 model). These two models allow the formation of a hexamer-DNA complex with no steric clash, which may operate in the co-regulation of the two genes *slr1738 *and *sll1621 *as shown for the 3 bp-spacing model in Figure 5. Detailed structural interactions between each dimer in these hexamer models are shown in Additional file 11 Figure S8. Schematic representation of the *sll1621*-*slr1738 *promoter region in these models predict that the binding of Slr1738 will preclude the binding of sigma factors, thereby impairing the transcription of the *slr1738 *and *sll1621 *genes. Furthermore, in this switch mechanism the two Slr1738 dimers bordering the hexameric regulator might be alternatively released in some environmental conditions thereby allowing the specific transcription of either *sll1621 *or *slr1738*. The second particular multimeric structure, possibly occurring in response to a high concentration of the FUR regulator [65,70], involves polymerization of multiple FUR proteins along their target DNA molecule which is thereafter no longer accessible to other enzymes. Such polymerization is only possible when a 6 bp-spacing (0-6-12 model) occurs between the FUR dimers (Figure 6). It could explain the structural organization of FUR-binding sites with repeated DNA sequence (GATAATGATAAT)_n_. By contrast, in the 0-3-6 hexameric model, this polymerization is not possible because a spacing of about 21 bp is necessary to allow the binding of the next hexamer onto the DNA helix.

## Conclusions

In the current post-genomic era the thousands of 3D protein structures available at the PDB database can be used as template to predict the possible fold of structurally uncharacterized proteins of biological interest. This task is important for mind-oriented analysis of the interactions between these proteins and their DNA and/or protein partners. In this frame, we used a combination of different types of data (structural, biochemical and genetic) to build and validate a relevant model of the tridimensional structure of a biologically important protein-DNA complex. This complex plays a central role in the regulation of cyanobacteria (environmentally crucial organisms) by interdependent oxidative and metal stresses. It is formed between the PerR/FUR-like Slr1738 regulator and its main DNA target, *i.e*. the promoter region of the divergent genes *slr1738 *and the peroxiredoxin (anti-oxidant) encoding gene *sll1621*. The detailed analysis of the Slr1738/DNA complex, and the related complex between genuine PerR/FUR-type regulators and their target DNA allowed us to better understand the interactions involved in the protein-DNA recognition and the stability/integrity of the structure.

The method we propose in this work to build the (Slr1738)_2_/DNA complex, that is based on fold-level similarity between DNA binding domains to transfer DNA orientation from a co-complex structure to a protein-only model, needs to be formalized and automated to DNA to be applied by other researchers to build other protein/DNA complexes of interest. We also develop a simple theoretical strategy to predict more complex structures involving the binding of multiple dimers of regulators to the same molecule of DNA. In this strategy, we consider each dimer as a molecular building block that can be moved along the target DNA molecule to hug it, and combine the resulting potential structure with footprinting and gel shift data to propose attractive model structures. One of these, a switch model, may explain a biological mechanism *i.e*. the presence/absence of coordinated expression of the genes co-regulated by the same Slr1738/FUR/PerR-type regulator, depending on the environmental conditions. We believe that our strategy for studying protein/DNA interactions will help to decipher the molecular basis of a wealth of regulatory mechanisms that are crucial for life.

## Competing interests

The authors declare that they have no competing interests.

## Authors' contributions

YB coordinated the project. YB, OD, FC and CC conceived and designed the experiments. PG and OD performed docking and analyzed the data. JYZ and CC carried out the molecular biology experiments. YB, OD and FC wrote the manuscript. All authors read and approved the final manuscript.

## Supplementary Material

Additional file 1 **Table S1**. Parameters of experimental RX structures used for the construction of the SLR-DNA complex. Click here for file

Additional file 2 **Figure S1**. Sequence alignment of the HTHw motif. Click here for file

Additional file 3 **Figure S2**. Schematic representation of two different protein-DNA enclosures involving a helix-turn-helix recognition motif. Click here for file

Additional file 4 **Table S2**. DNA-protein complexes parameters of the structures built with DNA template sequence. Click here for file

Additional file 5 **Figure S3**. Sequence of ahpC promoter region in cyanobacteria habouring a *Synechocystis-*like ahpC-Slr1738 intergenic organisation. Click here for file

Additional file 6 **Figure S4**. Determination of the transcription start site for the opposite genes *slr1738 *and *sll1621 *with the 5'-RACE technique. Click here for file

Additional file 7 **Figure S5**. Amino acids sequence alignments of the *Synechocystis *regulator Slr1738 with the protein from several cyanobacteria. Click here for file

Additional file 8 **Figure S6**. The different interactions that participate to the stabilization of the dimer interface of Slr1738. Click here for file

Additional file 9 **Figure S7**. FUR binding site motifs proposed in literature. Click here for file

Additional file 10 **Table S3**. Tetramer construction possibilities. Click here for file

Additional file 11 **Figure S8**. Interactions description in hexameric models. Click here for file
