# Supplementary material for: A transcriptional-switch model for Slr1738-controlled gene expression in the cyanobacterium Synechocystis
Source: BMC Struct Biol. 2012 Jan 30;12:1. doi: 10.1186/1472-6807-12-1 (PMC3293774; doi:10.1186/1472-6807-12-1)
Supplement: Additional file 1 — Table S1. Parameters of experimental RX structures used for the construction of the SLR-DNA complex. [file 1472-6807-12-1-S1.PDF]

Table S1: Parameters of experimental RX structures used for the construction of the SLR-DNA complex.

	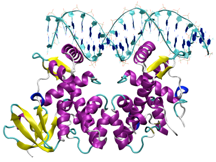	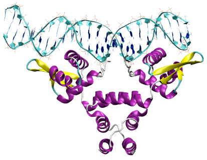	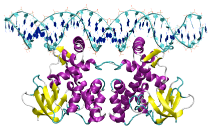	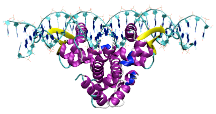
PDB code	1C0W	1SAX	1U8R	1Z9C
Specie	*Corynebacterium diphteriae*	*Staphylococcus aureus*	*Mycobacterium tuberculosis*	*Bacillus subtilis*
Experimental method & resolution (Å)	RX (3.2)	RX (2.8)	RX (2.75)	RX (2.64)
Protein monomer length (residues)	225	123	230	147
DNA length (bp)	21	24	32	28
Reference	Pohl et *al.* (1999)	Garcia-Castellans et *al.* (2004)	Wisedchaisri et *al.* (2004)	Hong et *al.* (2005)
