# Supplementary material for: A transcriptional-switch model for Slr1738-controlled gene expression in the cyanobacterium Synechocystis
Source: BMC Struct Biol. 2012 Jan 30;12:1. doi: 10.1186/1472-6807-12-1 (PMC3293774; doi:10.1186/1472-6807-12-1)
Supplement: Additional file 2 — Figure S1. Sequence alignment of the HTHw motif. [file 1472-6807-12-1-S2.PDF]

Figure S1: **Sequence alignment of the HTHw motif.** The sequences were aligned based on a serine at the beginning of the recognition helix. Identical and similar (S/T and I/L) amino acids presented in both Slr1738 and 1SAX are connected with a vertical line.

```
              helix  turn      helix        strand strand
SLR          QLLFDLQAAPTSSQATVYSSLKALQSVGLIREVLLEEGVCRY
                         |  |  | |  |
1SAX       ANNIIEEIQMQKDWSPKTIRTLITRLYKKGFIDRKKDNKIFQYYS
                         |
1Z9C          VKKMGEQLYLDSGTLTPMLKRMEQQGLITRKRSEEDERSVLISL
                         |
1C0W           RARIAERLEQSGPTVSQTVARMERDGLVVVASDRSLQM
                         |
1U8R           RARIAERLDQSGPTVSQTVSRMERDGLLRVAGDRHLEL
```
