# Supplementary material for: A transcriptional-switch model for Slr1738-controlled gene expression in the cyanobacterium Synechocystis
Source: BMC Struct Biol. 2012 Jan 30;12:1. doi: 10.1186/1472-6807-12-1 (PMC3293774; doi:10.1186/1472-6807-12-1)

Figure S2: Schematic representation of two different protein-DNA enclosures involving a helix-turn-helix recognition motif. (A) “Classical flat recognition” model where both recognition helices are parallel and contact the same side of the DNA molecule. (B) “FUR recognition model” where recognition helices are almost perpendicular and inserted in the opposite sides of the DNA molecule.

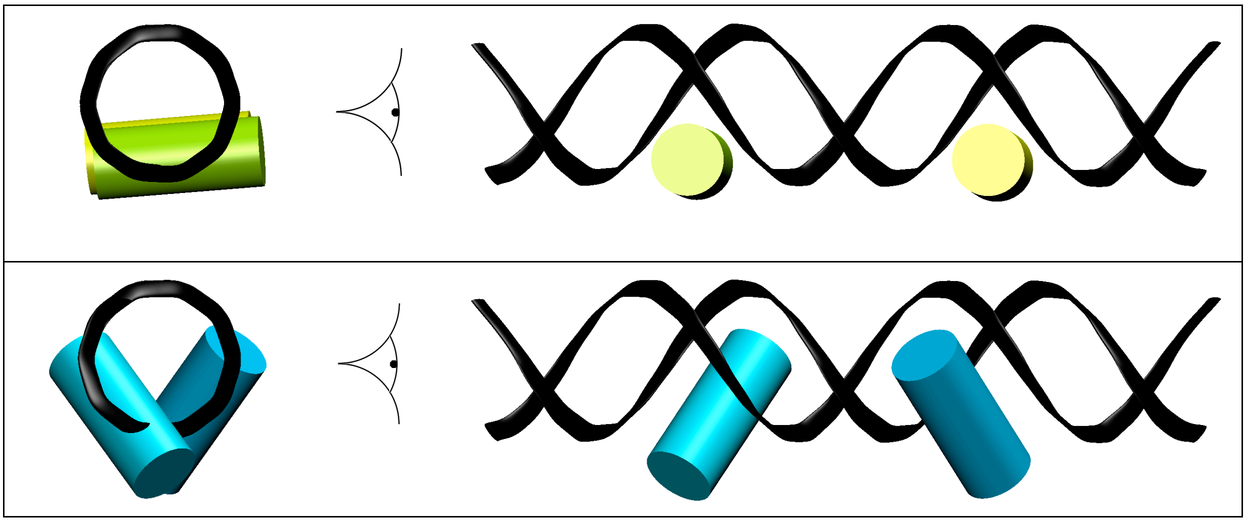

Supplement: Additional file 3 — Figure S2. Schematic representation of two different protein-DNA enclosures involving a helix-turn-helix recognition motif. [file 1472-6807-12-1-S3.PDF]
