# Supplementary material for: A transcriptional-switch model for Slr1738-controlled gene expression in the cyanobacterium Synechocystis
Source: BMC Struct Biol. 2012 Jan 30;12:1. doi: 10.1186/1472-6807-12-1 (PMC3293774; doi:10.1186/1472-6807-12-1)
Supplement: Additional file 4 — Table S2. DNA-protein complexes parameters of the structures built with DNA template sequence. [file 1472-6807-12-1-S4.PDF]

Table S2: DNA-protein complexes parameters of the structures built with DNA template sequence

Contact surfaces of 3D models obtained with different secondary structure motifs superpositions for the construction of DNA-$(Slr1738)_2$ complex. H: helix, S: strand.
Best values are in bold.

	Superposition type – Contact surface in $Å^2$			
PDB name	H4	H3H4	H4S1S2	H3H4S1S2
1C0W	867	816	**878**	855
1SAX	881	929	839	**944**
1U8R	874	869	**884**	851
1Z9C	976	863	**986**	909
