# Supplementary material for: A transcriptional-switch model for Slr1738-controlled gene expression in the cyanobacterium Synechocystis
Source: BMC Struct Biol. 2012 Jan 30;12:1. doi: 10.1186/1472-6807-12-1 (PMC3293774; doi:10.1186/1472-6807-12-1)

Figure S4: Determination of the transcription start site for the opposite genes *slr1738* and *sll1621* with the 5'-RACE technique. In each case, the nucleotide immediately downstream of the oligonucleotide primer (anchor) is the transcription start site (TSS indicated as +1, bent arrow) that was revealed by sequencing the RT-PCR cDNA products amplified with both the anchor and the respective *slr1738* (Panel A) and *sll1621* (Panel B) gene specific primers. The presumptive -35 and -10 promoter elements are written in bold cases and underlined while the presumptive Slr1738 DNA binding sequence are shaded in grey box (Panel C).

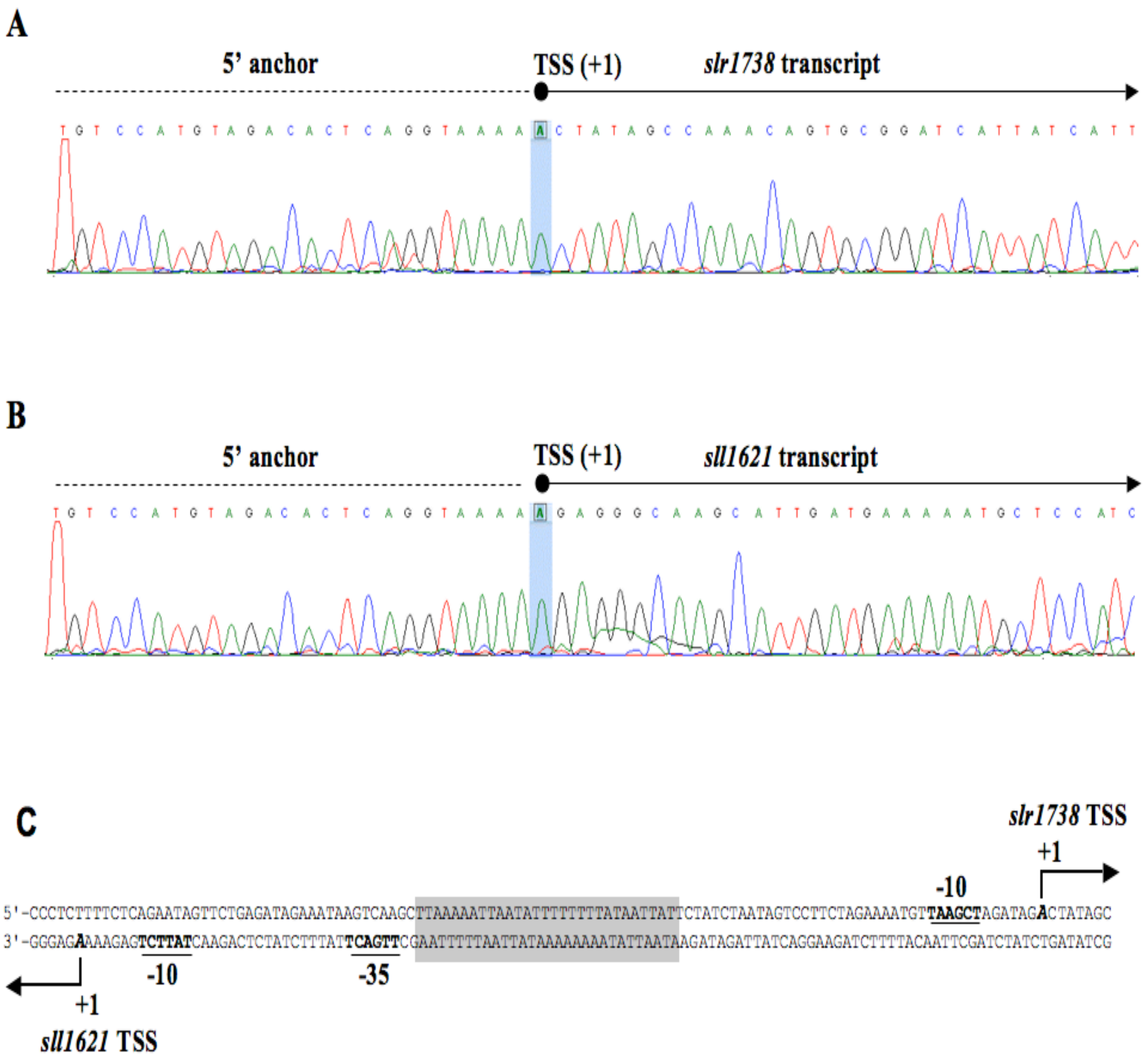

Supplement: Additional file 6 — Figure S4. Determination of the transcription start site for the opposite genes slr1738 and sll1621 with the 5'-RACE technique. [file 1472-6807-12-1-S6.PDF]
