# Supplementary material for: A transcriptional-switch model for Slr1738-controlled gene expression in the cyanobacterium Synechocystis
Source: BMC Struct Biol. 2012 Jan 30;12:1. doi: 10.1186/1472-6807-12-1 (PMC3293774; doi:10.1186/1472-6807-12-1)

Figure S5: Amino acids sequence alignments of the *Synechocystis* regulator Slr1738 with the protein from several cyanobacteria. (A), and the PerR or Fur regulators from various non cyanobacterial organisms (B).

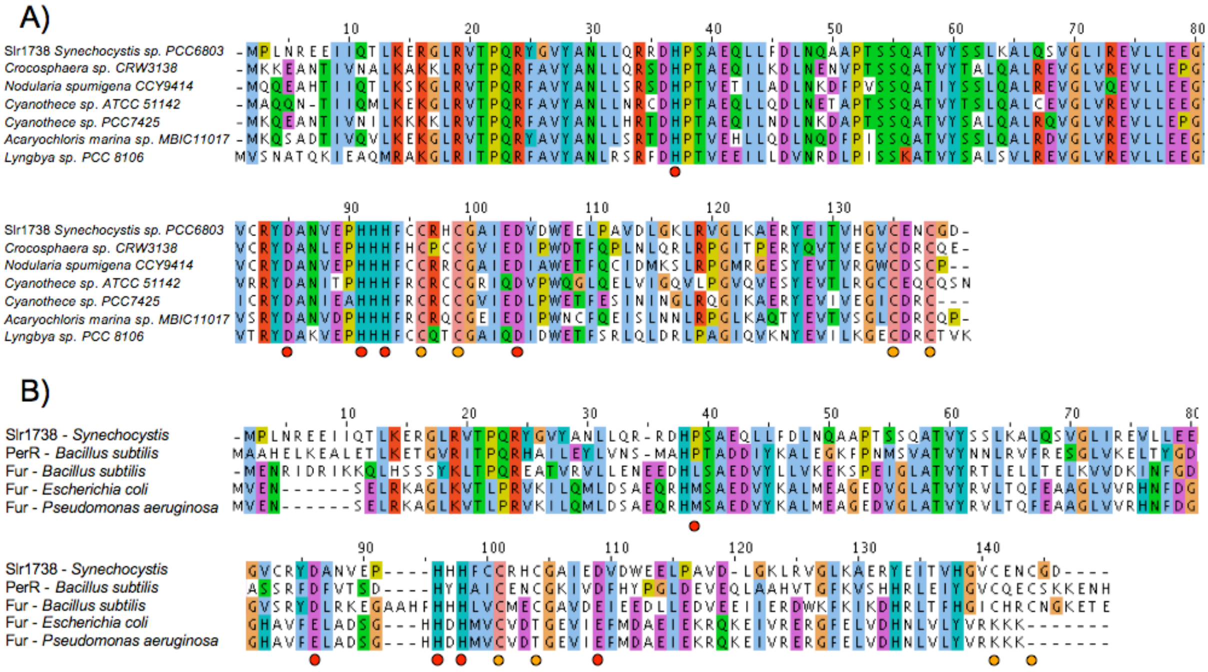

Supplement: Additional file 7 — Figure S5. Amino acids sequence alignments of the Synechocystis regulator Slr1738 with the protein from several cyanobacteria. [file 1472-6807-12-1-S7.PDF]
