# Supplementary material for: A transcriptional-switch model for Slr1738-controlled gene expression in the cyanobacterium Synechocystis
Source: BMC Struct Biol. 2012 Jan 30;12:1. doi: 10.1186/1472-6807-12-1 (PMC3293774; doi:10.1186/1472-6807-12-1)

Figure S6: The different interactions that participate to the stabilisation of the dimer interface of Slr1738. Hydrogen bonds (left); hydrophobic interactions (middle); salt bridges (right).

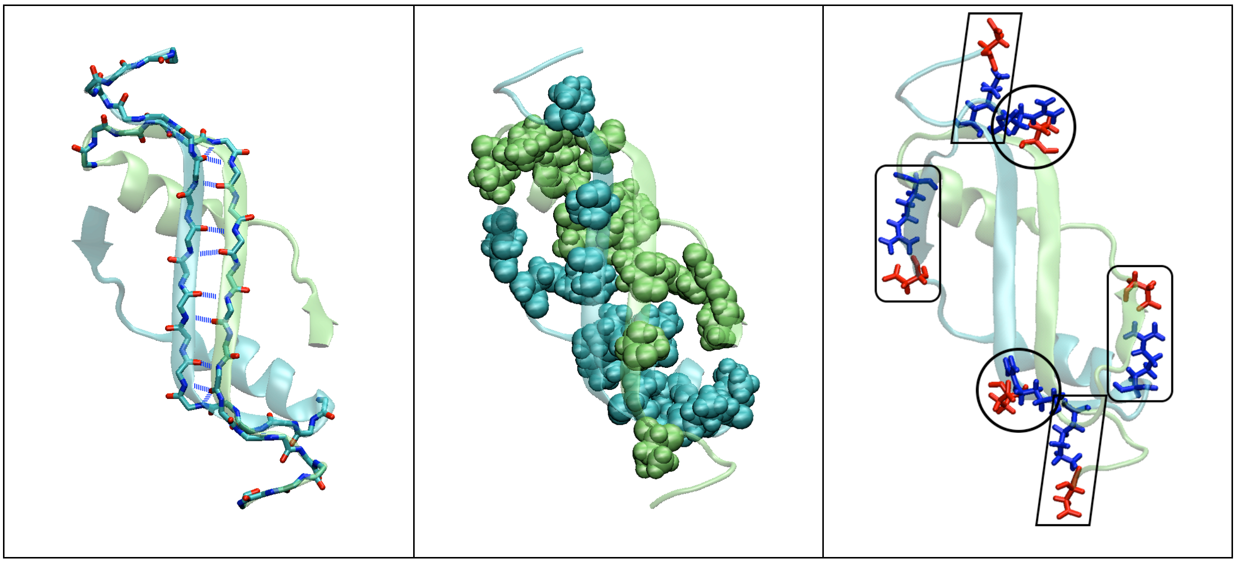

Supplement: Additional file 8 — Figure S6. The different interactions that participate to the stabilization of the dimer interface of Slr1738. [file 1472-6807-12-1-S8.PDF]
