# Supplementary material for: A transcriptional-switch model for Slr1738-controlled gene expression in the cyanobacterium Synechocystis
Source: BMC Struct Biol. 2012 Jan 30;12:1. doi: 10.1186/1472-6807-12-1 (PMC3293774; doi:10.1186/1472-6807-12-1)

Figure S7: FUR binding site motifs proposed in literature. (A) Classical 19 bp (9-1-9) inverted repeat sequence model [de Lorenzo et *al.*, 1987]. (B) The 7-1-7 model as described in [Baichoo and Helmann, 2002]. Interactions between both monomer A and B of Slr1738 and this model are shown in green and red respectively. (C) 18 bp model (6-6-6) which is simple addition of adjacent GATAAT hexamer unit [Escolar et *al.*, 1998].

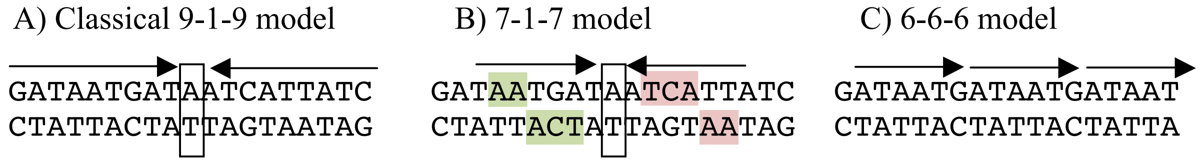

Supplement: Additional file 9 — Figure S7. FUR binding site motifs proposed in literature. [file 1472-6807-12-1-S9.PDF]
