# Supplementary material for: A transcriptional-switch model for Slr1738-controlled gene expression in the cyanobacterium Synechocystis
Source: BMC Struct Biol. 2012 Jan 30;12:1. doi: 10.1186/1472-6807-12-1 (PMC3293774; doi:10.1186/1472-6807-12-1)
Supplement: Additional file 10 — Table S3. Tetramer construction possibilities. [file 1472-6807-12-1-S10.PDF]

Table S3: **Tetramer construction possibilities.** The DNA molecule overlapped by Slr1738 protein is given both in base pair number and in surface (Å^2). CS: contact surface, bp: base pair.

offset (in bp)	1	2	**3**	4	5	**6**	7	8
CS (in A^2)	Clashes	Clashes	**1037**	0	0	**267**	806	Clashes
DNA overlapped	-	-	**28 (3831)**	29 (3800)	30 (3555)	**31 (3440)**	32 (3445)	-
offset (in bp)	9	10	11	**12**	13	14	15	16
CS (in A^2)	Clashes	Clashes	Clashes	**1477**	100	0	0	0
DNA overlapped	-	-	-	**37 (3596)**	38 (3483)	39	40	41 (3409)
offset (in bp)	17	18	19	20	21	22	23	24
CS (in A^2)	11	238	381	561	560	0	0	0
DNA overlapped	42	43	44 (3481)	45 (3297)	46 (3257)	47 (3282)	48	49
