# Supplementary material for: A transcriptional-switch model for Slr1738-controlled gene expression in the cyanobacterium Synechocystis
Source: BMC Struct Biol. 2012 Jan 30;12:1. doi: 10.1186/1472-6807-12-1 (PMC3293774; doi:10.1186/1472-6807-12-1)

Figure S8: **Interactions description in hexameric models**. Interactions between each dimer (d0 in lime, d3 in yellow, d6 in green and d12 in orange) occurring in the two hexameric models (0-3-6 and 0-6-12) of Slr1738. (A) tetramer 0-3, (B) tetramer 0-6, (c) tetramer 0-12. Electrostatic interactions are shown in red and blue colours and in wire representations whereas hydrophobic cores are shown in van der Waals representation mode. The numbers of the residues involved in interactions are shown with in subscript the monomer from which they belong and in highlight background colour the dimer in the hexamer model from which they belong.

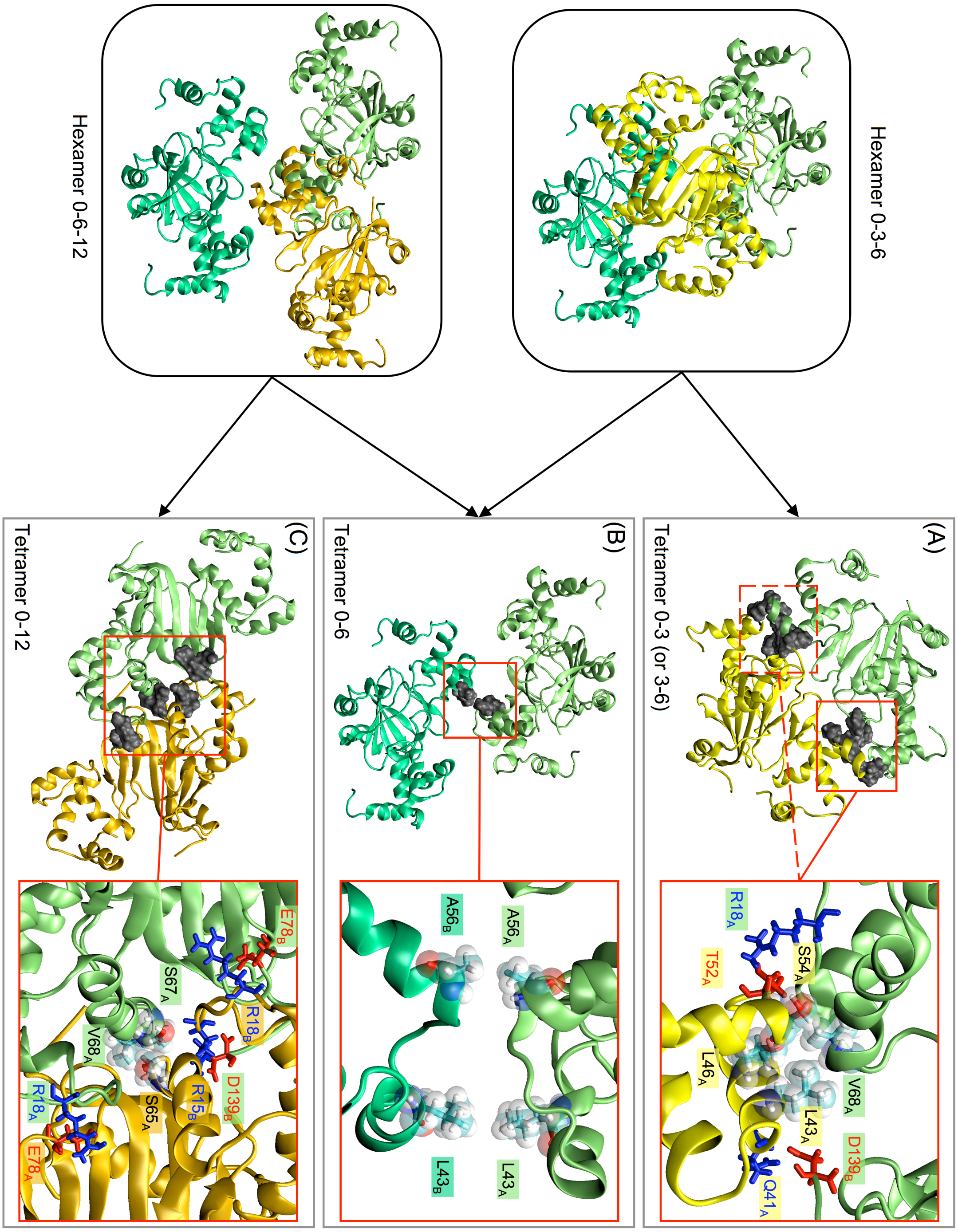

Supplement: Additional file 11 — Figure S8. Interactions description in hexameric models. [file 1472-6807-12-1-S11.PDF]
