# Supplementary material for: A transcriptional-switch model for Slr1738-controlled gene expression in the cyanobacterium Synechocystis
Source: BMC Struct Biol. 2012 Jan 30;12:1. doi: 10.1186/1472-6807-12-1 (PMC3293774; doi:10.1186/1472-6807-12-1)
Supplement: Additional file 5 — Figure S3. Sequence of ahpC promoter region in cyanobacteria habouring a Synechocystis-like ahpC-Slr1738 intergenic organisation. [file 1472-6807-12-1-S5.PDF]

Figure S3: Sequence of ahpC promoter region in cyanobacteria habouring a *Synechocystis*-like ahpC-Slr1738 intergenic organisation.

The A written in red case corresponds to the transcription start site of the *Synechocystis ahpC* gene we mapped in this study.

The -10 and -35 promoter boxes are written in bold and highlighted in grey

The position of the sequence are given from the translation start codon of ahpC)

Note the absence of consensus sequence in the AT-rich region (enlighted in blue) presently characterized.

		-35 box		-10 box			
-119	TTATATTAAGATTATTCAAGAAATTAAGTAATTATTACTG	**TTGACT**	TTTTCTTAGATAAGAAT	**TATTCT**	GAGATAAGACAGCAAAAGCT	-31	**Cyanothece sp. CCY0110**
-119	TTATATTAAGATTATTCAAGAAATTAGGTAATTATTACTG	**TTGACT**	TTTTCTTAAATAAGAAT	**TATTCT**	GAGATATAGCAGTAAAAGCT	-31	**Cyanothece sp. ATCC 51142**
-119	ATATCTTAAAATTACTCAATAAACTGAGTAGTTATTACCA	**TTGACT**	TTTTCTTAAATAAGAAT	**TATTCT**	GATATATGTTGGTAAAAACT	-31	**Crocosphaera watsonii WH 8501**
-148	TTAGATAGAATAATTATAAAAAAAAATATTAATTTTTAAGC	**TTGACT**	TATTTCTATCTCAGAAC	**TATTCT**	GAGAAA**A**GAGGGCAAGCATT	-59	**Synechocystis PCC6803**
-124	TATTTTATATCATAAAGAATTTTTTAAGTATATATTACTA	**TTGAAA**	TATTCTTAAATAAGAAT	**TATTCT**	TAGATATGTGGAGGCGATTG	-36	**Acaryochloris marina MBIC11017**
-107	TGAAATCAGAGTCATTCTGACAAATAGTTATAAATACTCA	**TTTACA**	AAAACACAAATCAGAAT	**TATTCT**	GAGTTAAGAACATTTTTAGT	-19	**Arthrospira platensis HZ01**
-126	TTATATGTGAACAAAACCAATTATTAAGTATTTATAACTA	**TTGAAT**	TTTTCGCATATCAGAAT	**TATTCT**	GATATAAGTGAGCAGAACGC	-38	**Nodularia spumigena CCY9414**
